# Revealing a Wetting–Penetration–Interlocking Mechanism for the Interfacial Reinforcement of Degradable Liquid Plugs via Silane-Induced Microstructure Engineering

**DOI:** 10.3390/polym17121660

**Published:** 2025-06-15

**Authors:** Yuexin Tian, Yintao Liu, Haifeng Dong, Xiangjun Liu, Jinjun Huang

**Affiliations:** 1 Petroleum Engineering Technology Institute of Southwest Petroleum Branch, SINOPEC, Deyang 618000, China; liuyintao.xnyq@sinopec.com (Y.L.); donghaifeng.xnyq@sinopec.com (H.D.); 2 State Key Laboratory of Oil and Gas Reservoir Geology and Exploitation, Southwest Petroleum University, Chengdu 610500, China; liuxiangjun@swpu.edu.cn (X.L.); huangjinjun@swpu.edu.cn (J.H.)

**Keywords:** liquid plug, interfacial modification, octadecyltrichlorosilane (OTS), bonding strength enhancement, multiscale synergy mechanism

## Abstract

Hypothesis: Poor interfacial bonding and wetting incompatibility limit the performance of degradable liquid plugs under high-pressure conditions. It is hypothesized that silane-induced interfacial engineering can build a multiscale structure that enhances adhesion via coupled wetting, penetration, and interlocking mechanisms. Experiments: A C18 silane-modified steel surface was constructed and tested for its bonding behavior with an epoxy-based degradable plug. Interfacial strength, compressive capacity, and microstructure were analyzed using mechanical tests, SEM, AFM, and contact angle measurements. Surface energy was calculated via the Owens–Wendt model. Findings: The silane-treated interface exhibited a significant enhancement in interfacial bonding strength (up to 445%) and shear strength (73.8% increase), attributed to the formation of a 391.6 nm thick infiltrated interlayer and strong chemical anchoring (Si–O–Fe bonds). Contact angle decreased from 74.0° to 53.6°, with interfacial energy increasing by 26.2%, confirming improved wettability and energy compatibility. A triadic enhancement pathway of “wetting–penetration–interlocking” was established, supported by microstructural imaging and theoretical modeling. This work provides mechanistic insights and practical guidance for the design of robust liquid plug systems in complex wellbore environments.

## 1. Introduction

Degradable liquid plugs have emerged as a promising alternative to traditional packers for temporary zonal isolation and flow control in complex wellbore environments. These materials offer adaptable sealing behavior, tunable degradation rates, and compatibility with intelligent completion systems, enabling their application in horizontal wells and multistage hydraulic fracturing operations [1,2,3]. However, their long-term sealing integrity is frequently compromised by poor interfacial bonding with metallic casing surfaces, especially under high differential pressures and thermal stress gradients [4,5,6].

Although attention has been paid to the mechanical robustness and degradation behavior of these systems, failure at the plug–steel interface remains the dominant failure mode. Specifically, the interface is vulnerable due to (i) low surface energy and poor wettability on the steel substrate, (ii) inadequate chemical anchoring during curing, and (iii) insufficient structural continuity to resist crack propagation under downhole stresses [7,8,9,10].

Several strategies have been proposed to enhance plug–steel adhesion, including polymer matrix modifications, surface roughening, and the application of commercial silane coupling agents [2,4]. While these methods offer partial improvements, they often fail to address all three of these core challenges simultaneously. Recent studies have emphasized the need to bridge the gap between interfacial chemistry and macroscopic performance. For example, Tsuji et al. [11] reported that the spatial distribution and molecular interactions of curing agents significantly influence epoxy–metal bonding. Similarly, Van Dam et al. [12] demonstrated that durable adhesion requires the co-optimization of surface roughness and chemical composition.

Despite these efforts, existing models typically treat interfacial phenomena in isolation, lacking a unified framework. Although the Owens–Wendt model has been widely used to characterize interfacial free energy [13,14], its integration with interfacial penetration behavior, anchoring mechanisms, and fracture resistance in soft solid systems like liquid plugs remains underexplored. The absence of a multiscale mechanistic model connecting thermodynamic wettability, nanostructural infiltration, and interfacial energy dissipation fundamentally limits predictive design capabilities.

To address these gaps, this study introduces a silane-induced interfacial engineering strategy using long-chain alkyltrichlorosilanes (C18 OTS). We hypothesize that this approach enables the in situ formation of a multifunctional interphase governed by a triadic reinforcement mechanism:

Wetting activation → Nanoscale penetration → Mechanical interlocking.

This pathway not only facilitates strong Si–O–Fe covalent bonding but also creates a deeply infiltrated, flexible interphase with enhanced compatibility and energy dissipation capacity. Recent work by Bechikh et al. [15] has shown that tailored silane structures can effectively modulate surface energy and form entangled anchoring networks that improve fracture resistance.

To validate this mechanism, we integrate

- Contact angle measurements and Owens–Wendt-based surface energy analysis;
- Interfacial morphology characterization via SEM and AFM;
- Quantitative evaluation of bonding, shear, and compressive strength;
- A theoretical penetration and anchoring model.

While previous studies have demonstrated the interfacial reinforcing effects of silane coupling agents via mechanisms such as chemical bonding (e.g., Si–O–Fe or Si–O–Si; van Dam et al. [12]), hydrogen bonding and surface bridging (Uetsuji et al. [16]), or plasma-induced surface activation (Jung et al. [17]), most of these systems are limited to single-mode enhancement. Moreover, the structural evolution and mechanical coupling across different length scales have rarely been explored. In this study, we propose a triadic mechanism of wetting–penetration–interlocking (WPI), where long-chain OTS molecules not only enhance interfacial wettability and energy compatibility but also induce capillary-driven penetration and microstructural interlocking with the steel substrate. This integrated path enables a robust and scalable enhancement strategy for degradable liquid plugs under complex wellbore conditions.

## 2. Materials and Methods

### 2.1. Materials

The epoxy matrix was prepared using epoxidized soybean oil-modified epoxy resin (industrial grade, Shandong Delan New Materials Technology Co., Ltd., Shandong, China). Methyl tetrahydrophthalic anhydride (MTHPA, 99%) and 2,4,6-tris(dimethylaminomethyl)phenol (DMP-30, 98%) were purchased from Tianjin Kemiou Chemical Reagent Co., Ltd., Tianjin, China and Shanghai Macklin Biochemical Co., Ltd., Shanghai, China, respectively. Dicumyl peroxide (DCP, 95%) and polyethylene glycol diacrylate (PEGDA, 98%) were obtained from Changzhou Ruixu Chemical Co., Ltd., Chengdu, China and Chengdu Huaan New Materials Co., Ltd., Chengdu, China. Octadecyltrichlorosilane (OTS, 97%) was supplied by Shanghai Yuanye Bio-Technology Co., Ltd., Shanghai, China. All solvents used, including n-hexane and acetone, were of analytical grade.

Steel substrates (N80 casing-grade, 50 mm × 50 mm × 3 mm) were cut and polished using 600-grit sandpaper and ultrasonically cleaned in acetone for 30 min before use.

### 2.2. Instruments

The following instruments were employed in this study: HH-SJ magnetic oil bath (Changzhou Guoyu Instrument Manufacturing Co., Ltd., Changzhou, China), C3003 precision balance (Hangzhou Want Balance Instrument Co., Ltd., Hangzhou, China), DJ1C-60 high-torque stirrer (Jiangsu Jincheng Guosheng Laboratory Instrument Factory, Changzhou, China), and DFG101-3 thermostatic oven (Shangyu Huyue Instrument Equipment Factory, Shangyu, China).

Mechanical tests were performed using an SHT4106 electrohydraulic servo universal testing machine (MTS Industrial Systems Co., Ltd., Shenzhen, China) and a WDW-100E universal compression tester (Jinan New Era Testing Equipment Co., Ltd., Jinan, China). Morphological characterization was conducted using an XL30 SEM (FEI, now part of Thermo Fisher Scientific, Hillsboro, OR, USA) and Dimension ICON AFM (Bruker Corporation, Billerica, MA, USA). Contact angles were measured using a JC2000D contact angle goniometer (Shanghai Zhongchen Digital Technic Apparatus Co., Ltd., Shanghai, China).

### 2.3. Specimen Preparation and Surface Modification

#### 2.3.1. Preparation of Liquid Plug Formulation

The liquid plug was formulated using a mass ratio of 50:50:1:1.5:0.5 for epoxy resin, MTHPA, DMP-30, DCP, and PEGDA. The components were added to a magnetic stirring oil bath (HH-SJ) and mixed at 80 °C for 15 min. After degassing, the mixture was poured into custom PTFE molds (thickness controlled at 3 mm) and cured at 80 °C for 4 h. Specimens were demolded and aged at ambient temperature (25 °C) for 24 h prior to testing.

#### 2.3.2. OTS Surface Modification of Steel Substrates

Steel substrates were polished with 600-grit sandpaper, ultrasonically cleaned in acetone for 30 min, and dried at 60 °C. OTS was dissolved in n-hexane at 0.5%, 1.0%, and 1.5% concentrations. The cleaned plates were immersed in OTS solution for 30 min, followed by 20 min of air exposure for hydrolysis, and thermal treatment at 80 °C for 1 h to induce Si–O–Fe bonding.

#### 2.3.3. Fabrication of Interfacial Shear Specimens

A double-plate lap shear configuration was used. OTS-treated steel plates served as the bottom substrate. The pre-degassed plug formulation was injected and spread within a PTFE spacer mold (3 mm gap), then covered by a second clean steel plate. After curing at 80 °C for 4 h and ambient aging for 24 h, the specimens were ready for shear testing.

### 2.4. Interfacial Shear Strength Test

Shear tests were performed on an SHT4106 electrohydraulic servo testing machine. Specimens (50 mm × 50 mm bonded area, 3 mm thickness) were tested at a rate of 1.0 mm/min. Dimensions were verified using a digital caliper (±2% tolerance). Shear strength was calculated using Equation (1):

(1) $$\tau =\frac{F_{\max}}{A}$$

where *F_max_* is the maximum load at failure (N), and *A* is the bonded area (mm^2^).

For each OTS group, five specimens were tested (*n* = 5), and the average ± SD was reported. Fracture patterns were recorded for failure mode analysis.

### 2.5. Interfacial Bonding Strength Test

Cylindrical steel molds (50 mm diameter × 100 mm height) were filled with 100 g of the plug formulation and cured at 120 °C for 24 h. A 49 mm diameter plunger applied axial force at 0.5 mm/min. Bonding strength was calculated according to Equation (2):

(2) $$p=\frac{10F_{s}}{\pi hD}$$

where *p* is the longitudinal interfacial bonding strength (MPa), *F_S_* is the peak load (kN), and *S_C_* is the cylindrical contact area (cm^2^), calculated as *π* × *D* × *h*, where *D* is the outer diameter (cm) and h is the height (cm).

All measurements were conducted in triplicate (*n* = 3), and real-time load–displacement curves were recorded.

### 2.6. Compressive Strength Test

Compressive tests were conducted on cylindrical specimens (25 mm × 25 mm) using a WDW-100E tester at 1 mm/min. Compressive strength was calculated by Equation (3):

(3) $$\sigma_{c}=\frac{F_{max}}{A}$$

where σ*_c_* is the compressive strength (MPa), *F_max_* is the peak compressive load (N), and *A* is the loaded area (mm^2^). Each test was repeated three times (*n* = 3).

### 2.7. Microstructural Characterization

Push-out fracture specimens were prepared to expose the plug–steel interface. SEM imaging was conducted using a JSM-IT500 (JEOL Ltd., Tokyo, Japan) after cold-mounting, grinding, and sputtering. Features such as interfacial tearing, penetration structures, and micro-anchoring were analyzed.

AFM scanning (5 μm × 5 μm, Dimension ICON) was used to assess surface topography and interfacial layer thickness. Morphological changes across different OTS concentrations were visualized to support mechanism interpretation.

### 2.8. Contact Angle Measurement

Static water contact angles were measured using a JC2000D goniometer (Shanghai Zhongchen Digital Technic Apparatus Co., Ltd., Shanghai, China) at 25 ± 1 °C and 45–55% RH. A 5 μL droplet was dispensed on each sample. For each steel plate, three sites were measured three times (*n* = 9). Surface energy was calculated using the Owens–Wendt method with water and diiodomethane as probe liquids. All samples were dried in a desiccator for 12 h before testing.

## 3. Results and Discussion

### 3.1. Interfacial Mechanical Enhancement

**Figure 1:**
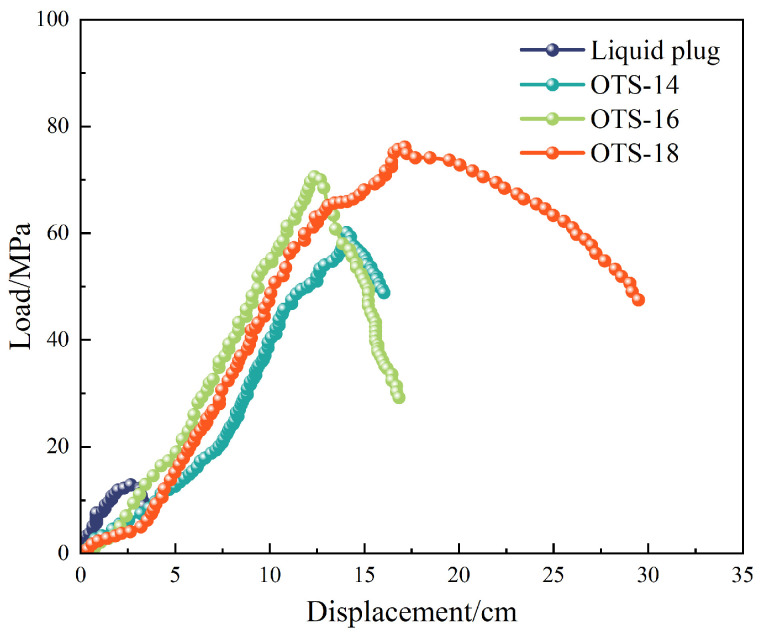
Effect of OTS molecular structure on interfacial bonding strength.

To evaluate the mechanical enhancement effects of silane modification at the liquid plug–steel interface, a systematic study was conducted by varying the molecular chain length and surface concentration of octadecyltrichlorosilane (OTS). Three silane agents with increasing alkyl chain length—C14 (OTS-14), C16 (OTS-16), and C18 (OTS-18)—were compared. As shown in Figure 1 and Table 1, the bonding strength increased progressively from 8.7 MPa to 11.0 MPa with longer chains, highlighting the synergistic contribution of covalent anchoring via Si–O–Fe bonds and hydrophobic chain entanglement with the polymer matrix. Based on this superior performance, OTS-18 was selected for subsequent concentration-dependent studies and interfacial mechanism investigations. Longer chains facilitated denser molecular packing and stronger interfacial cooperation, promoting stress dissipation and crack resistance.

**Figure 2:**
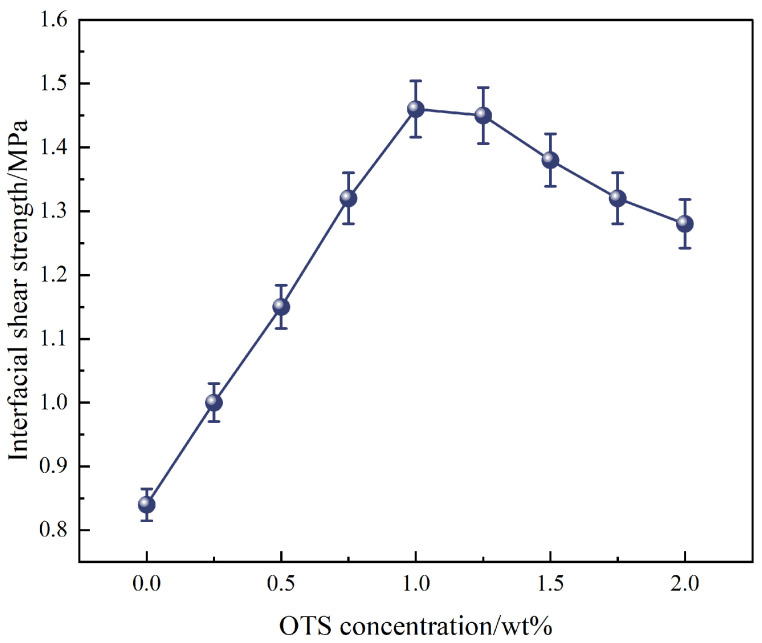
Effect of OTS-18 concentration on interfacial shear strength.

**Figure 3:**
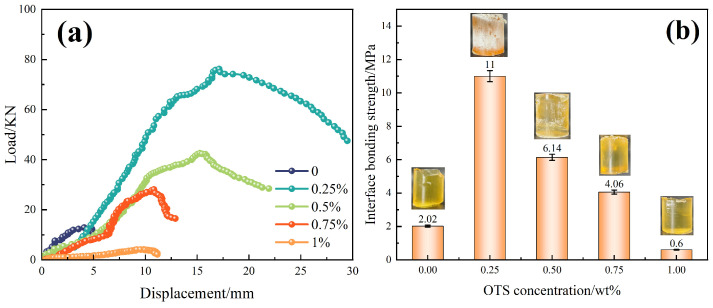
Effect of OTS-18 on interfacial bonding strength. (**a**) Load–displacement curves. (**b**) Bonding strength versus OTS-18 concentration.

Further investigation using OTS-18 revealed a concentration-dependent trend in both interfacial shear strength and bonding strength. As shown in Figure 2, shear strength increased from 0.84 MPa to a maximum of 1.46 MPa at 1.0 wt% OTS-18 (a 73.8% increase), then declined slightly at 2.0 wt% due to excessive surface coverage leading to disordered packing and wetting inhomogeneity. Similarly, bonding strength peaked at 11.0 MPa at 0.25 wt%, a 445% increase over the unmodified sample, but dropped markedly at higher concentrations (Figure 3b), indicating a concentration-threshold effect. The load–displacement curves in Figure 3a show a transition from brittle to ductile failure at optimal OTS-18 content, confirming enhanced stress transfer and strain tolerance at the interface.

**Figure 4:**
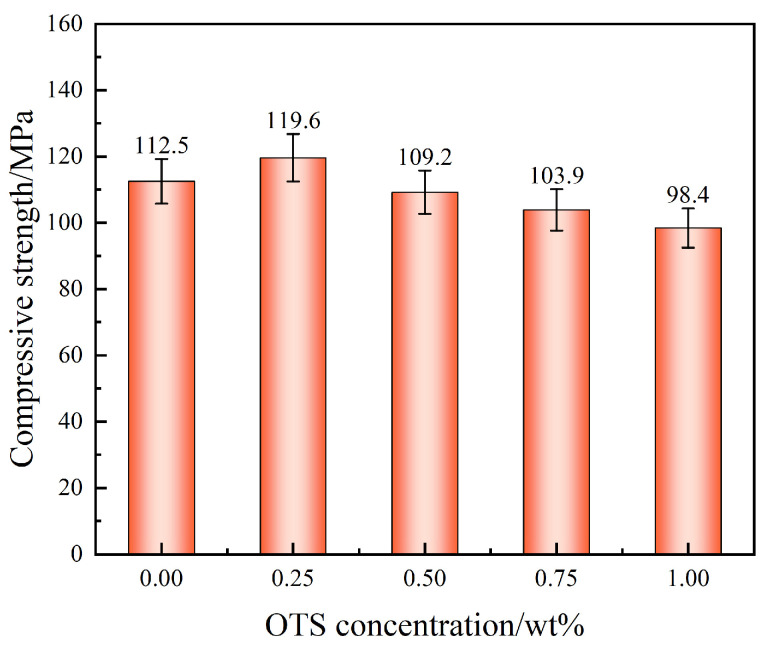
Effect of OTS-18 concentration on the compressive strength of the liquid plug.

In addition to interfacial adhesion, the intrinsic compressive strength of the liquid plug was also evaluated to assess bulk mechanical stability. As presented in Figure 4, compressive strength improved slightly from 112.5 MPa (control) to 119.6 MPa at 0.25 wt% OTS-18 (+6.3%), attributed to enhanced network compactness and structural cohesion. However, higher OTS-18 concentrations reduced compressive strength due to internal phase separation and interference with the epoxy crosslinking process.

**Figure 5:**
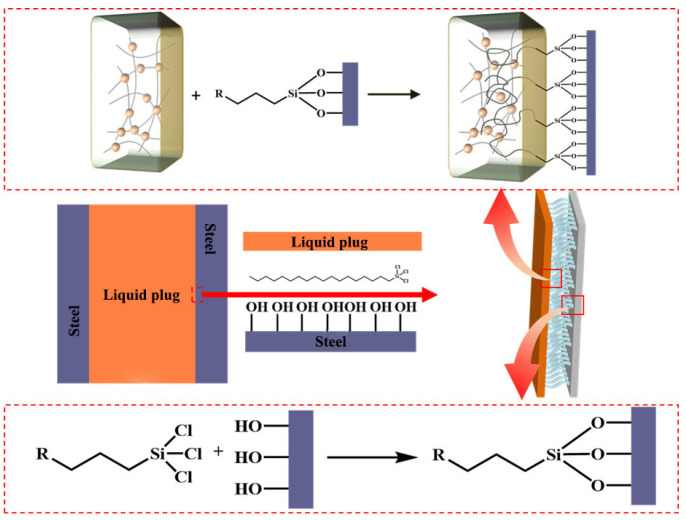
Schematic of the interfacial mechanism of OTS in the liquid plug–steel system. The orange block represents the liquid plug, and the purple block represents the steel substrate. Red arrows indicate the magnified views of the interface regions marked by red boxes.

The combined results support a multiscale interfacial enhancement mechanism, illustrated in Figure 5: (i) trichlorosilane anchoring forms robust chemical bonds with the steel surface; (ii) flexible alkyl chains entangle with the polymer matrix to form a semi-interpenetrating network; (iii) microstructural compatibility promotes energy dissipation and crack resistance. An optimal OTS-18 concentration of 0.25–1.0 wt% is recommended to balance interfacial reinforcement and bulk stability. These findings provide mechanistic insights and design guidance for tailoring silane coupling agents in degradable plug systems under complex downhole conditions.

### 3.2. Microstructural Mechanism Analysis

**Figure 6:**
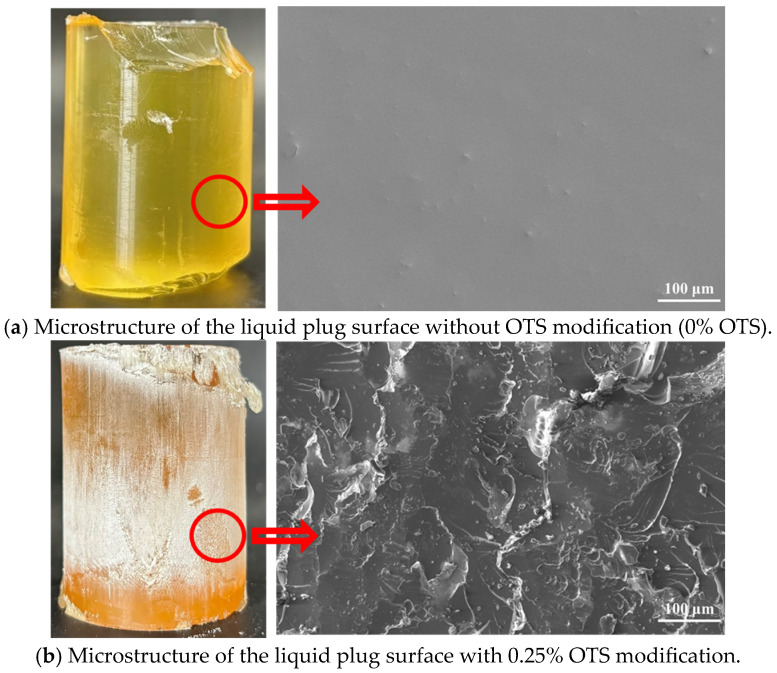
Interfacial fracture morphology of liquid plugs ((**a**) unmodified; (**b**) 0.25 wt% OTS-18 modified, The red circle and arrow highlight the selected surface region and its corresponding magnified SEM image).

To elucidate the microstructural origin of enhanced interfacial bonding between the liquid plug and steel surface, a multiscale characterization approach integrating SEM, AFM, contact angle measurement, and interfacial energy analysis was employed. As shown in Figure 6, the unmodified sample (0% OTS) exhibited smooth fracture surfaces with no tearing features or shear deformation, indicative of weak adhesion and brittle failure (Figure 6a). In contrast, the 0.25 wt% OTS-modified interface revealed rough morphologies, tearing pits, and crack deflection patterns, suggesting effective mechanical interlocking and energy dissipation (Figure 6b).

**Figure 7:**
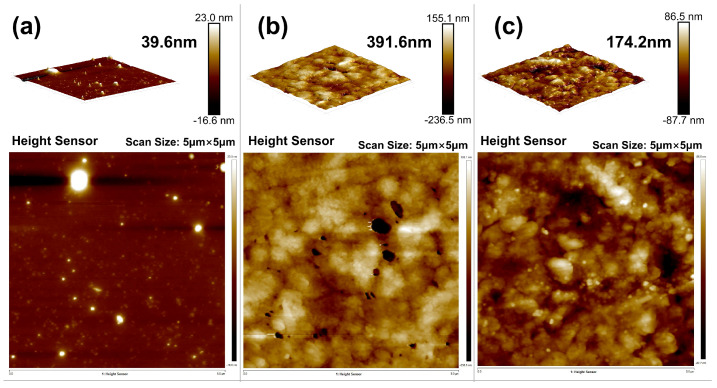
AFM characterization of interfacial bonding layers: (**a**) 0% OTS-18 (thickness: 39.6 nm); (**b**) 0.25% OTS-18 (thickness: 391.6 nm); (**c**) 0.5% OTS-18 (thickness: 174.2 nm).

AFM scans provided further insights into interfacial layer evolution. The untreated sample showed a thin, poorly developed bonding layer (~39.6 nm), while the OTS-modified interface formed a continuous polymer-silane layer with a thickness up to 391.6 nm (Figure 7). This nearly tenfold increase confirmed effective molecular infiltration and entanglement at optimal OTS concentration. At higher concentrations (0.5–1.0 wt%), interfacial thickness decreased and undulations appeared, implying phase separation and localized aggregation that disrupted uniform bonding.

**Figure 8:**
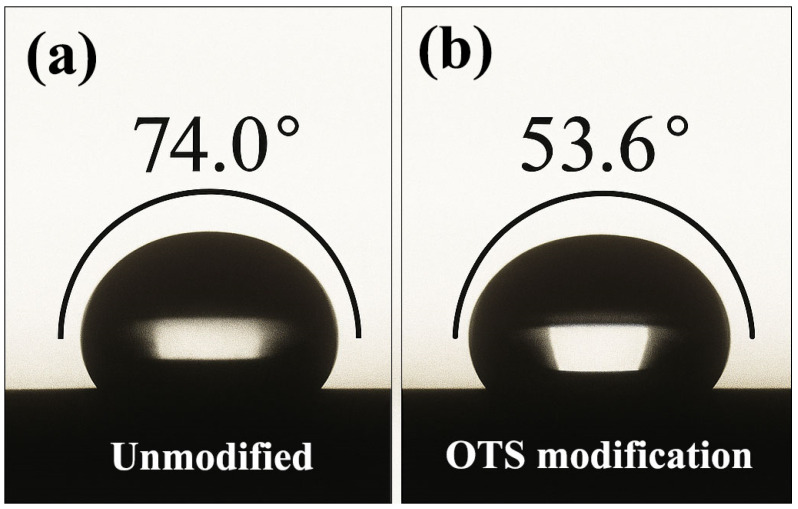
Contact angle variations of liquid plug on steel surface before and after OTS-18 modification ((**a**) unmodified; (**b**) 1.0 wt% OTS-18-modified).

**Figure 9:**
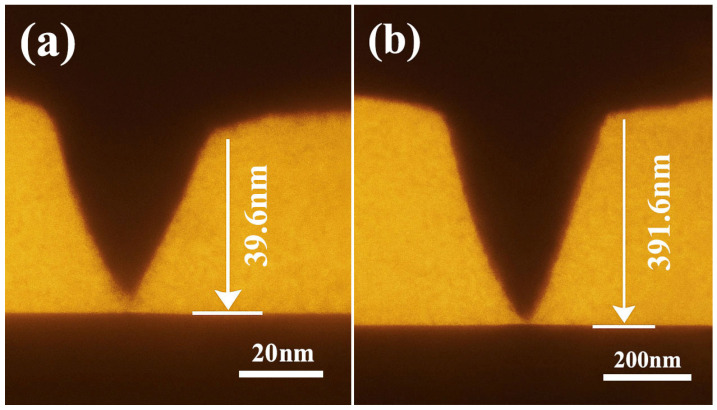
Cross-sectional morphology of infiltration depth at the liquid plug–steel interface ((**a**) unmodified; penetration depth: 39.6 nm; scale bar: 20 nm; (**b**) 1 wt% OTS-18-modified; penetration depth: 391.6 nm; scale bar: 200 nm).

To assess wettability and infiltration behavior, contact angle measurements were conducted (Figure 8). The unmodified plug exhibited a high contact angle of 78.5°, reflecting poor wetting on the steel surface. After OTS treatment, the contact angle decreased to 32.8°, accompanied by a significant increase in surface energy (from 32.1 to 40.5 mJ·m^−2^, Table 2), particularly in the polar component. This thermodynamically favored condition facilitated deeper plug infiltration into surface micropores, as confirmed in cross-sectional morphology (Figure 9).

**Figure 10:**
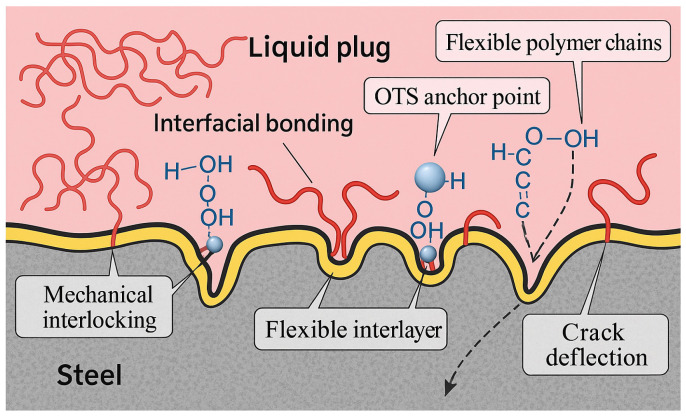
Schematic diagram of interfacial reinforcement mechanisms at the liquid plug–steel interface. The gray region represents the steel substrate, the pink area denotes the bulk phase of the liquid plug, and the yellow layer indicates the flexible interfacial interlayer induced by OTS modification. Red lines correspond to flexible polymer chains, and blue spheres represent hydroxyl groups involved in hydrogen bonding interactions.

Based on these observations, a multiscale reinforcement mechanism is proposed (Figure 10):

(1) OTS molecules form covalent Si–O–Fe bonds via terminal SiCl_3_ groups, enhancing chemical anchoring;

(2) Long alkyl chains entangle with the resin matrix, forming a flexible interfacial nanolayer that buffers stress;

(3) Interfacial topography evolves into root-like protrusions and interlocking zones, deflecting crack propagation and dissipating strain energy.

This synergistic mechanism integrates chemical bonding, physical interlocking, and wetting-induced infiltration to create a robust interfacial architecture. The formation of a ~400 nm thick energy dissipation layer improves fracture resistance and stress distribution, ultimately ensuring interfacial integrity under complex downhole conditions. These insights lay the groundwork for molecular-level interface design in degradable sealing systems.

### 3.3. Thermo-Temporal Effects on Interfacial Bonding Performance

**Figure 11:**
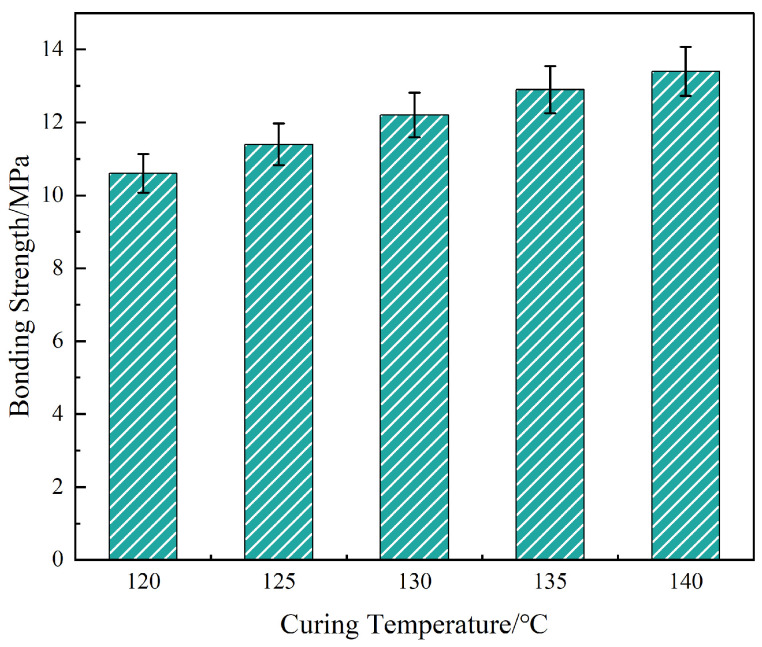
Effect of curing temperature on interfacial bonding strength of the liquid plug system.

**Figure 12:**
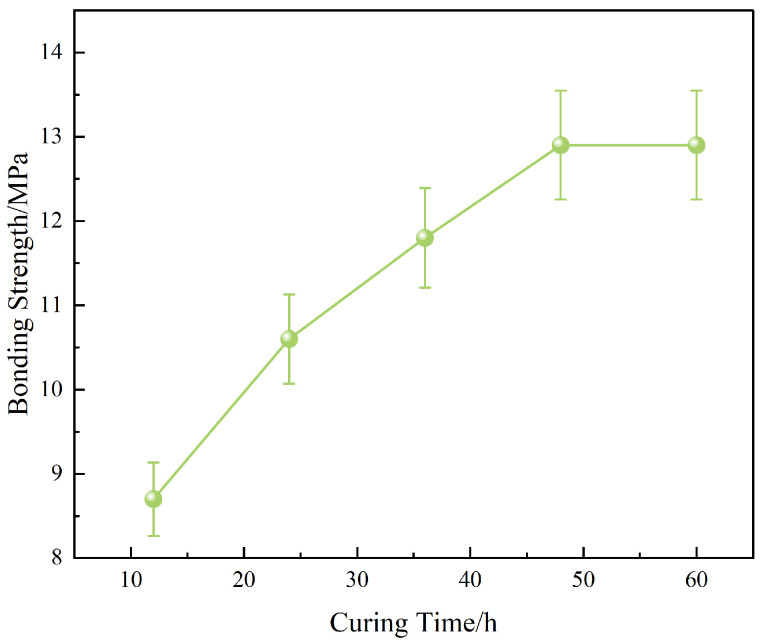
Effect of curing time on interfacial bonding strength.

To further optimize the interfacial bonding performance of OTS-modified liquid plugs, the thermo-temporal parameters of the curing process were systematically evaluated. The bonding strength was measured across a curing temperature range of 120–140 °C and durations of 12–60 h, under a constant OTS-18 concentration of 0.25 wt%. Results are presented in Figure 11 and Figure 12.

As shown in Figure 11, interfacial bonding strength increased from 10.6 MPa at 120 °C to 13.4 MPa at 140 °C, with the steepest improvement (15.1%) observed between 120 and 130 °C. This enhancement correlates with two concurrent phenomena: (i) elevated temperatures accelerate ring-opening polymerization between epoxidized soybean oil and anhydride, thereby increasing crosslink density and modulus of the bulk matrix; (ii) OTS condensation with steel surface hydroxyls is promoted, forming a denser interfacial nanolayer. These effects collectively enhance cohesive and adhesive strength.

However, the strength gain diminished beyond 135 °C. This plateau is attributed to thermally induced mismatch in expansion coefficients between the steel and cured resin, leading to microvoids and residual stresses that disrupt local interfacial continuity. This suggests a thermal performance threshold above which the benefits of enhanced reactivity are negated by internal mechanical stress.

Time-dependent analysis (Figure 12) further revealed a progressive increase in bonding strength from 8.7 MPa (12 h) to 12.9 MPa (48 h), with a near saturation plateau thereafter. This trend indicates that the curing reaction and interfacial anchoring via OTS molecules reach equilibrium within 48 h. Prolonged curing (60 h) provided negligible additional benefit, suggesting a practical optimization point for field applications.

These results support a dual-phase regulation mechanism: temperature accelerates reaction kinetics and interfacial covalent bonding; time ensures network consolidation and anchoring completion. Importantly, the thermal–temporal coupling also enhances interfacial penetration depth (as supported by Figure 7) and reduces defect generation during stress transfer. Therefore, a curing condition of 130–135 °C for 48 h is recommended to achieve maximal bonding performance while maintaining microstructural integrity and operational feasibility.

### 3.4. Theoretical Mechanism Derivation and Multiscale Enhancement Pathway Construction

**Figure 13:**
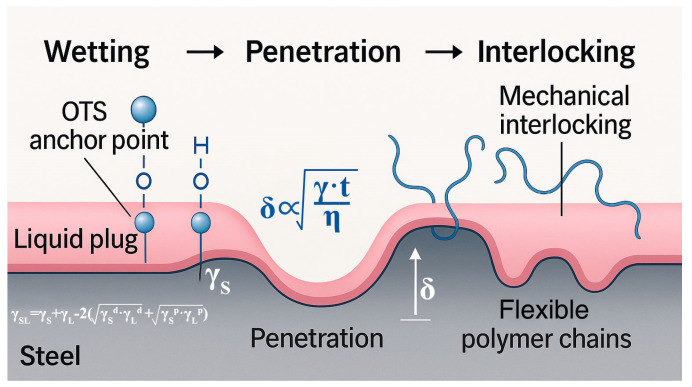
Theoretical enhancement model of the liquid plug interface under OTS-induced wetting–penetration–coupling effects. The pink region represents the liquid plug matrix, and the gray area denotes the steel substrate. Blue polymer chains illustrate the penetration and interlocking behavior of flexible polymer segments into the steel surface microstructure.

To elucidate the mechanism by which OTS modification enhances the interfacial bonding strength of the liquid plug system, a three-step theoretical framework is proposed, namely, wetting enhancement → penetration enhancement → coupling enhancement, as schematically illustrated in Figure 13.

Upon OTS addition, the interfacial wettability is significantly improved, as evidenced by the reduction in contact angle (θ) and corresponding increase in interfacial free energy. The enhanced spreading behavior of the liquid plug on the steel substrate can be quantitatively described by the Owens–Wendt model, where the solid–liquid interfacial tension (*γ_SL_*) is given as follows:

(4) $$\gamma_{SL}=\gamma_{S}+\gamma_{L}-2\left(\sqrt{\gamma_{S}^{d}\cdot \gamma_{L}^{d}}+\sqrt{\gamma_{S}^{p}\cdot \gamma_{L}^{p}}\right)$$

In Equation (5), *γ_S_* and *γ_L_* represent the total surface energy of the solid (steel) and the liquid (plug), respectively; *γ^d^* and *γ^p^* denote the dispersive and polar components, respectively.

The improved wettability facilitates deeper penetration of the liquid plug into surface depressions and microvoids prior to gelation, forming a thicker anchoring layer (δ). This process can be simplified as a capillary-driven diffusion mechanism, where the penetration depth δ is dependent on surface energy (γ), viscosity (η), and curing time (t), as given by:

(5) $$\delta \propto \sqrt{\frac{\gamma \cdot t}{\eta }}$$

Furthermore, the long alkyl chains of OTS self-assemble at the interface, forming a flexible, entangled nanolayer that physically interlocks with the epoxy matrix. This hybrid layer serves to both dissipate interfacial stress and impede crack propagation along the steel–polymer interface. Collectively, these phenomena constitute a triadic “wetting–penetration–interlocking” pathway, coherently explaining the experimentally observed enhancements in shear strength, bonding strength, compressive resistance, and penetration depth.

**Figure 14:**
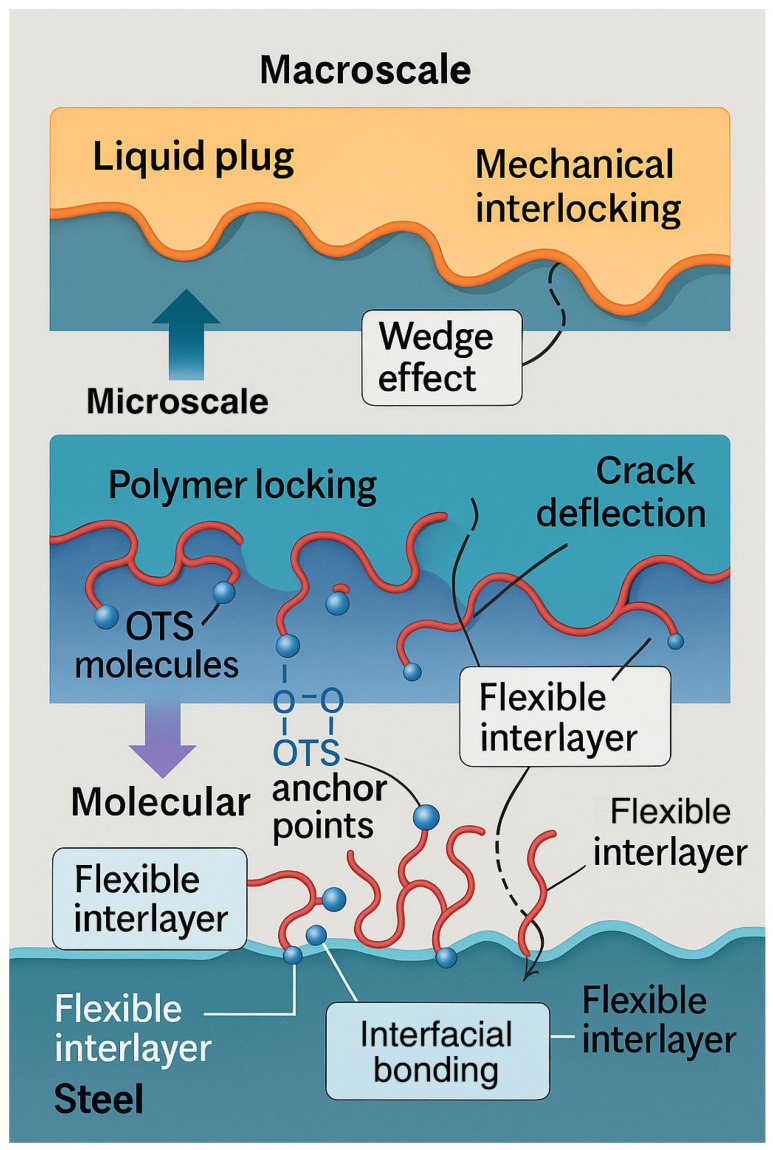
Schematic diagram of multiscale reinforcement pathway.

Integrating the quantitative improvements in bonding performance (Section 3.1), microstructural evolution (Section 3.2), and thermal–temporal regulation (Section 3.3), a comprehensive multiscale interfacial reinforcement model is proposed, as shown in Figure 14. This framework unifies the synergistic effects at the molecular, microstructural, and macroscopic scales.

At the molecular level, OTS molecules undergo in situ hydrolysis and condensation with hydroxylated steel surfaces, forming robust –Si–O–Fe covalent bonds that serve as primary anchoring points. Contact angle measurements showed a substantial reduction from 74.0° to 53.6°, reflecting enhanced surface energy compatibility. The calculated interfacial free energy further confirms stronger thermodynamic affinity between the liquid plug and the steel substrate.

At the microstructural level, thermal curing enables the formation of a flexible interphase layer, which penetrates into the steel’s surface microtextures and promotes mechanical interlocking. SEM and AFM analyses (Figure 6, Figure 7 and Figure 9) reveal a significant increase in roughness and interfacial layer thickness—from 39.6 nm (unmodified) to 391.6 nm (0.25 wt% OTS). Moreover, the observed crack deflection and branching confirm enhanced energy dissipation and complex fracture pathways.

At the macroscopic scale, mechanical performance is comprehensively improved. At 0.25 wt% OTS, the interfacial bonding strength reaches 11.0 MPa and compressive strength rises to 119.6 MPa (Figure 3 and Figure 4). These enhancements are attributed to the synergistic effects of molecular anchoring, nanostructural entanglement, and stress-buffering interphases. In summary, the OTS-induced interfacial reinforcement follows a hierarchically structured wetting–penetration–interlocking pathway, forming a continuous enhancement cascade from molecular activation to structural entrapment and ultimately to macroscopic performance improvement. This model provides a robust theoretical foundation for interfacial design in degradable sealing systems subjected to complex downhole environments.

To contextualize the novelty and scope of the proposed mechanism, a comparative summary of representative silane-modified interfacial enhancement strategies is provided in Table 3. Unlike previous models that typically emphasize either chemical bonding or surface roughness alone, the present study introduces a hierarchically integrated mechanism involving sequential wetting activation, capillary-driven penetration, and mechanical interlocking. This multiscale strategy not only forms a physically measurable interfacial layer (~391.6 nm) but also leads to superior enhancements in bonding strength (↑445%) and shear performance (↑73.8%), outperforming conventional silane systems. The table highlights these distinctions in terms of structural depth, dominant mechanisms, and performance gains.

### 3.5. Limitations and Future Research

#### 3.5.1. Experimental Scope and Characterization Limitations

Although the proposed OTS-induced interfacial reinforcement mechanism demonstrates significant improvements in bonding performance, several limitations in experimental design and scope should be acknowledged, along with directions for future work.

First, the material selection in this study was limited to N80 casing steel, a common substrate in conventional wellbore applications. However, other downhole environments often involve high-alloy or corrosion-resistant steels such as 13Cr or P110. The applicability of the proposed triadic mechanism to these materials remains to be validated through future tests involving diverse alloy compositions and surface chemistries.

Second, all experiments were conducted under standard laboratory conditions, without simulating high-pressure, high-temperature (HPHT), or chemically aggressive environments such as brine or acidic media. Although curing and strength evaluations were performed at elevated temperatures (up to 120 °C), comprehensive environmental testing is required to assess the durability of the silane-modified interface under realistic wellbore conditions.

Third, due to the exploratory nature of this work, all mechanical results were based on small sample sets (*n* = 3). While standard deviation bars have been added in Figure 2, Figure 3, Figure 4, Figure 11 and Figure 12 to reflect data variability, no statistical significance testing (e.g., ANOVA or *p*-values) was applied. Increasing the number of replicates and incorporating hypothesis testing in future studies will improve the reliability of performance evaluations.

Fourth, although this study demonstrates a triadic enhancement mechanism involving wetting, penetration, and interlocking, the relative contributions of each factor remain difficult to isolate experimentally. Control studies—for instance, modifying surface energy without altering roughness, or vice versa—were not conducted. Nevertheless, based on the observed results, it is qualitatively inferred that enhanced wettability facilitates initial contact, capillary-driven penetration promotes the formation of a thicker anchoring layer (~391.6 nm), and interlocking structures contribute to stress dissipation and crack deflection. Future work will include variable-decoupled designs to quantify the individual impact of each mechanism.

Fifth, limitations in characterization techniques also constrain interpretation. Only two groups (0 and 0.25 wt% OTS) were included in the contact angle tests, restricting the ability to construct a continuous wetting behavior profile. Moreover, although SEM and AFM analyses supported the presence of anchoring layers, the use of similar magnifications across samples with an order-of-magnitude difference in penetration depth (39.6 nm vs. 391.6 nm) may lead to misrepresentations. Follow-up studies will incorporate full-range contact angle mapping and scale-adjusted imaging to improve visual clarity and analytical accuracy.

Additionally, direct spectroscopic or thickness characterization of the silane-modified layer (e.g., via XPS, FTIR, or ellipsometry) was not conducted due to equipment limitations. Evidence of OTS grafting was inferred indirectly through contact angle changes, AFM topography, and mechanical performance. Future investigations will employ advanced surface analysis techniques to validate molecular bonding and interfacial architecture more rigorously.

Finally, although the study identifies an optimal OTS concentration range of 0.25–1.0 wt%, the robustness of this formulation under fine-gradient changes or variable curing conditions remains untested. While the consistency of results within this range suggests formulation resilience, tolerance testing is needed to define a reliable operational window. High-resolution parametric studies in future work will help guide field-scale implementation and formulation control under varying downhole conditions.

#### 3.5.2. Industrial Scalability and Environmental Considerations

Although the OTS-modified liquid plug demonstrated promising interfacial reinforcement and controllable degradability in laboratory-scale experiments, its scalability and operational feasibility remain subject to several considerations. From a field deployment perspective, the system’s two-component formulation—comprising the epoxy–anhydride matrix and trace OTS modifier—offers logistical simplicity and compatibility with existing mixing and injection units commonly used in wellbore operations. Preliminary assessments showed acceptable storage stability at 25 °C for over 30 days, but long-term shelf life under varying temperature and humidity conditions requires further validation.

Regarding silane handling, OTS is sensitive to moisture and requires appropriate sealing during storage and mixing. In field operations, this demands basic containment protocols (e.g., nitrogen sealing or desiccant inclusion), which are standard practices for reactive liquid components and do not pose substantial logistical challenges.

From an environmental perspective, the degradation byproducts of the plug system—primarily low-molecular-weight organic acids and silanols—are inherently biodegradable or hydrolyzable under downhole temperature and water-rich conditions. However, their cumulative ecological impacts (e.g., on groundwater or soil microbiota) remain to be systematically assessed. Future work will involve controlled-release testing, field pilot studies, and life-cycle impact analyses to ensure the system’s environmental compatibility and industrial scalability.

## 4. Conclusions

(1) This study demonstrates that octadecyltrichlorosilane (OTS-18), serving as a bifunctional interfacial modifier, significantly enhances the interfacial bonding performance of liquid plugs on steel substrates. At an optimal concentration of 0.25 wt%, OTS-18 achieved a 445% increase in bonding strength and a 73.8% improvement in shear strength relative to the unmodified system. The results highlight the critical influence of alkyl chain length on silane–substrate coupling efficiency and offer molecular-level design guidance for sealing applications.

(2) Interfacial structural analysis confirms that OTS molecules promote the formation of a flexible anchoring layer and root-like mechanical interlocks at the steel interface, governed by a synergistic “anchoring–entanglement–buffering” mechanism. The interfacial penetration depth increased from 39.6 nm to 391.6 nm, enabling enhanced stress transfer and crack deflection. These findings validate a multiscale reinforcement pathway characterized by wetting activation, penetration-driven interlocking, and nanoscale stress dissipation.

(3) A coupled structure–property framework was established by integrating interfacial wettability (contact angle and surface energy), nanoscale infiltration (AFM-measured interfacial layer thickness), and mechanical strength performance (shear and bonding strength). In comparison to previously reported silane-based systems that often rely on singular reinforcement mechanisms, such as chemical anchoring, hydrogen bonding, or surface roughening, the present work constructs a hierarchically coupled “wetting–penetration–interlocking” (WPI) pathway. This model uniquely enables the formation of a measurable ~391.6 nm interfacial dissipation layer and achieves simultaneous enhancements of 445% in bonding strength and 73.8% in shear performance. These results demonstrate that the WPI mechanism not only bridges molecular interactions with structural features but also offers a scalable strategy for high-integrity sealing design under complex downhole conditions.

## Figures and Tables

**Table 1:** Chemical structures of interface modification agents and their bonding strength. Grey, white, purple, and green spheres represent carbon (C), hydrogen (H), silicon (Si), and chlorine (Cl) atoms, respectively.

OTS	Molecular Structure	Bonding Strength/MPa
OTS-14	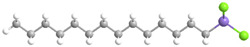	8.7
OTS-16	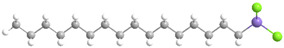	10.2
OTS-18	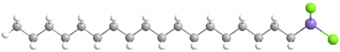	11.0

**Table 2:** Surface free energy parameters of steel interface before and after 1 wt% OTS-18 modification (Owens–Wendt model [18]).

Surface Condition	Contact Angle (°)	Total Surface Energy γ_s_ (mJ/m^2^)	Dispersive Component γ_s_^d^ (mJ/m^2^)	Polar Component γ_s_^p^ (mJ/m^2^)
Unmodified surface	74.0	32.1	25.3	6.8
1 wt% OTS-18-modified surface	53.6	40.5	29.7	10.8

**Table 3:** Comparison of representative silane-modified interfacial reinforcement models and proposed WPI mechanism.

Study	Silane Type	Reported Mechanism	Structural Depth	Performance Improvement	Multiscale Coupling
van Dam et al. [12]	γ-GPS	Si–O–Fe bonding + roughness	Not reported	Adhesion ↑ ~80%	✕
Uetsuji et al. [16]	APTES	H-bond bridging + surface activation	Nanoscale layer	Adhesion ↑ ~40%	Partial
Joseph et al. [19]	GPS (w/ heat)	Condensation + thermal curing	Interfacial gel zone	Adhesion ↑ ~60%	✕
Jung et al. [17]	Alkoxysilane + DBD	Plasma-induced Si–O–Si/C–O hybrid	0.1–0.5 µm	Shear ↑ ~120%	✔
Chen et al. [20]	Mixed silane	Wettability + filler matrix anchoring	Micropore level	Adhesion ↑ ~2×	Partial
**This work**	OTS (C18)	Wetting → Penetration → Interlocking	**~391.6 nm**	**Adhesion ↑ 445%** **Shear ↑ 73.8%**	**✔✔✔ Full**

Note: Structural depth and performance improvement are based on reported AFM/SEM results and bonding/shear strength comparisons under similar conditions. ↑ indicates enhancement relative to the unmodified control; ✕ means no evidence of multiscale coupling; ✔ indicates partial coupling; ✔✔✔ denotes full multiscale coupling verified by hierarchical structural response. Bold text indicates the data corresponding to this work for emphasis and comparison.

## Data Availability

The original contributions presented in this study are included in the article. Further inquiries can be directed to the corresponding author.
